# Supplementary material for: Million-year solar wind irradiation recorded in chang’E-5 and chang’E-6 samples
Source: Nat Commun. 2025 Oct 16;16:9197. doi: 10.1038/s41467-025-64239-8 (PMC12533252; doi:10.1038/s41467-025-64239-8)
Supplement: Supplementary file 1 — Supplementary Information [file 41467_2025_64239_MOESM1_ESM.pdf]

Supplementary information for Million-year Solar Wind Irradiation Recorded in Chang'E-5 and Chang'E-6 Samples

AUTHORS: Renrui Liu[1, 2†], Xiaoping Zhang[1, 2*], Sizhe Zhao[1, 2†], Yi Xu[1, 2*], Pengwei Luo[1, 2†], Yang Li[3*], Xiaojia Zeng[1, 3], Chenkun Sun[1, 2], Ronghua Pang[3], Chen Li[3, 4], Xiongyao Li[3], Lianghai Xie[5], Zhiguo Meng[1, 6], Qiugang Zong[1, 2], Chi Wang[5]

Affiliations:

[1]State Key Laboratory of Lunar and Planetary Sciences, Macau University of Science and Technology, Macau, China.

[2]CNSA Macau Center for Space Exploration and Science, Macau, China.

[3]Center for Lunar and Planetary Sciences, Institute of Geochemistry, Chinese Academy of Sciences, Guiyang, China.

[4]School of Engineering, Yunnan University, Kunming, China.

[5]State Key Laboratory of Space Weather, National Space Science Center, Chinese Academy of Sciences, Beijing, China.

[6]College of Geoexploration Science and Technology, Jilin University, Changchun, China.

† These authors contributed equally to this work.

* Corresponding author. Email: xpzhangnju@gmail.com yixu@must.edu.mo liyang@mail.gyig.ac.cn

Table S1. Exposure age and SW-damaged rim thickness of lunar soil grain.

Grain Number	Exposure Age (Myr)	SW-damaged Rim Thickness (nm)
CE5-PL1	0.77±0.06	60.70±2.07
CE5-PL2	0.56±0.04	39.91±1.64
CE5-PL3	1.22±0.12	57.43±5.20
CE5-PL4	1.14±0.08	64.05±3.33
CE5-PL5	0.81±0.07	51.11±3.86
CE5-PL6	1.11±0.09	47.89±1.45
CE5-PL7	0.96±0.08	59.66±1.29
CE6-PL1	2.57±0.22	73.70±3.08
CE6-PL2	0.96±0.06	19.02±1.60
CE6-PL3	1.1±0.09	59.24±2.58
CE6-PL4	2.4±0.13	62.81±4.52
CE6-PL5	1.2±0.06	53.33±3.69
CE6-PL6	1.22±0.15	50.24±6.15
CE6-PL7	3.18±0.17	108.54±1.63
CE6-PL8	1.93±0.11	74.36±4.96

Table S2. EDS analysis of particle An and mineral types.

Grain Number	An	Mineral
CE5-PL1	90	Anorthite
CE5-PL2	93	Anorthite
CE5-PL3	91	Anorthite
CE5-PL4	88	Bytownite
CE5-PL5	87	Bytownite
CE5-PL6	83	Bytownite
CE5-PL7	82	Bytownite
CE6-PL1	92	Anorthite
CE6-PL2	91	Anorthite
CE6-PL3	91	Anorthite
CE6-PL4	86	Bytownite
CE6-PL5	89	Bytownite
CE6-PL6	89	Bytownite
CE6-PL7	81	Bytownite
CE6-PL8	86	Bytownite

Table S3. Impact of injected ion parameters on damage depth and vacancy generation rate. SRIM simulations reveal that as the incident energy of injected ions increases, both the damage depth (penetration depth into the material) and the vacancy generation rate (number of vacancies per ion) rise.

Injection Energy (keV)	Incident Angle (Degree)	Damage Depth (nm)	Vacancy Generation Rate (Vacancies/Ion)
0.3	0.0	12.0	0.3
1.0	0.0	30.3	1.8
3.0	0.0	70.9	4.2

Table S4. SRIM-simulated damage depths of Fe and H ions implanted into bytownite ($Na_{0.3}Ca_{0.7}Al_{1.3}Si_{2.7}O_8$, density 2.72 g/cm^3) and anorthite ($CaAl_2Si_2O_8$, density 2.75 g/cm^3). For a given ion and implantation energy, the damage depths are nearly identical between the two minerals.

	Injected ion: Fe Damage depth (μm)	
Ion Energy (MeV/nucleon)	Bytownite	Anorthite
0.107143	3.2838	3.2876
0.116071	3.4173	3.4212
0.125000	3.5504	3.5543
0.142857	3.7965	3.8005
0.160714	4.0314	4.0355
0.178571	4.2355	4.2396
0.196429	4.4390	4.4432
0.214286	4.6320	4.6363
0.232143	4.8047	4.8090
0.250000	4.9872	4.9914
0.267857	5.1494	5.1537
0.285714	5.3114	5.3157
0.303571	5.4733	5.4776
0.321429	5.6350	5.6393
0.357143	5.9396	5.9439
0.401786	6.3055	6.3098
0.446429	6.6609	6.6651
0.491071	7.0058	7.0100
0.535714	7.3504	7.3445
0.580357	7.6747	7.6788
0.625000	8.0088	8.0028
0.669643	8.3327	8.3267
0.714286	8.6464	8.6404
0.803571	9.2888	9.2726
0.892857	9.9104	9.8939
0.982143	10.5313	10.5147
1.071429	11.1517	11.1249
1.160714	11.7617	11.7347
1.250000	12.3613	12.3342
1.428571	13.5955	13.5479
1.607143	14.8072	14.7590
1.785714	16.0369	15.9782
1.964286	17.2551	17.1860

	Injected ion: H Damage depth (nm)	
Ion Energy (keV)	Bytownite	Anorthite
0.100	5.4	5.5
0.110	5.8	5.9
0.120	6.2	6.3
0.130	6.5	6.6
0.140	6.9	6.9
0.150	7.3	7.3
0.160	7.6	7.6
0.170	8.0	8.0
0.180	8.3	8.3
0.200	9.0	8.9
0.225	9.7	9.7
0.250	10.5	10.5
0.275	11.3	11.3
0.300	12.1	12.1
0.325	12.8	12.8
0.350	13.6	13.5
0.375	14.2	14.2
0.400	15.0	15.0
0.450	16.4	16.4
0.500	17.7	17.8
0.550	19.1	19.2
0.600	20.4	20.5
0.650	21.8	21.8
0.700	23.0	23.1
0.800	25.6	25.6
0.900	28.1	28.1
1.000	30.5	30.5
1.100	32.9	32.9
1.200	35.3	35.3
1.300	37.6	37.5
1.400	39.9	39.8
1.500	42.0	42.0
1.600	44.3	44.1

2.142857	18.4820	18.4025
2.321429	19.7079	19.6181
2.500000	20.9532	20.8531
2.678571	22.2081	22.0977
2.857143	23.4725	23.3618
3.035714	24.7465	24.6256
3.214286	26.0403	25.9191
3.571429	28.7195	28.5674
4.017857	32.1676	31.9943
4.464286	35.7100	35.5100
4.910714	39.3400	39.1100
5.357143	43.0700	42.8300
5.803571	46.9100	46.6400
6.250000	50.8600	50.5600
6.696429	54.9000	54.5800
7.142857	59.0600	58.7100
8.035714	67.9100	67.4800
8.928571	77.1600	76.6600
9.821429	86.8500	86.2800
10.000000	88.7800	88.2000

1.700	46.4	46.4
1.800	48.5	48.5
2.000	52.7	52.6
2.250	57.8	57.6
2.500	62.7	62.6
2.750	67.6	67.5
3.000	72.3	72.2
3.250	77.0	76.9
3.500	81.6	81.4
3.750	86.0	85.9
4.000	90.4	90.3
4.500	98.9	98.9
5.000	107.2	107.1
5.500	115.2	115.1
6.000	123.0	122.9
6.500	130.6	130.6
7.000	137.9	138.0
8.000	152.1	152.1
9.000	165.7	165.7
10.000	178.8	178.7

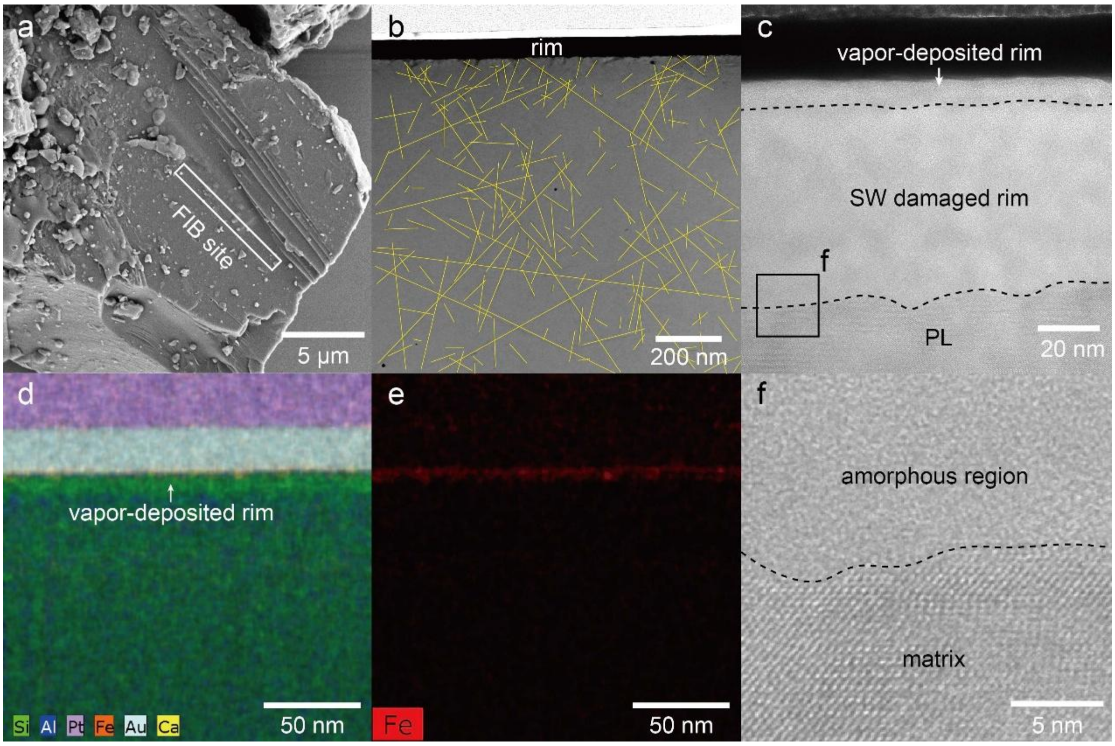

Fig. S1. (a) Secondary electron (SE) image of the studied CE5-PL1 grain. The white square indicates the focused ion beam (FIB) cross-section site. (b-c, f) Scanning transmission electron microscope (STEM) and high-resolution transmission electron microscopy (HRTEM) image of the FIB cross-section surface region. The black dashed line represents the boundary between the amorphous rim and the matrix. The rim includes both the vapor-deposited rim and the solar wind damaged rim. The solar energetic particle tracks within the matrix are marked by yellow solid lines. (d-e) Quantitative transmission electron microscopy-energy dispersive X-ray spectroscopy (TEM-EDS) composition maps (Au, Pt, Al, Si, Ca, Fe). The composition shows that the vapor-deposited rim is rich in iron, while the composition of the solar wind damaged rim is consistent with the matrix. SW, solar wind; PL, plagioclase.

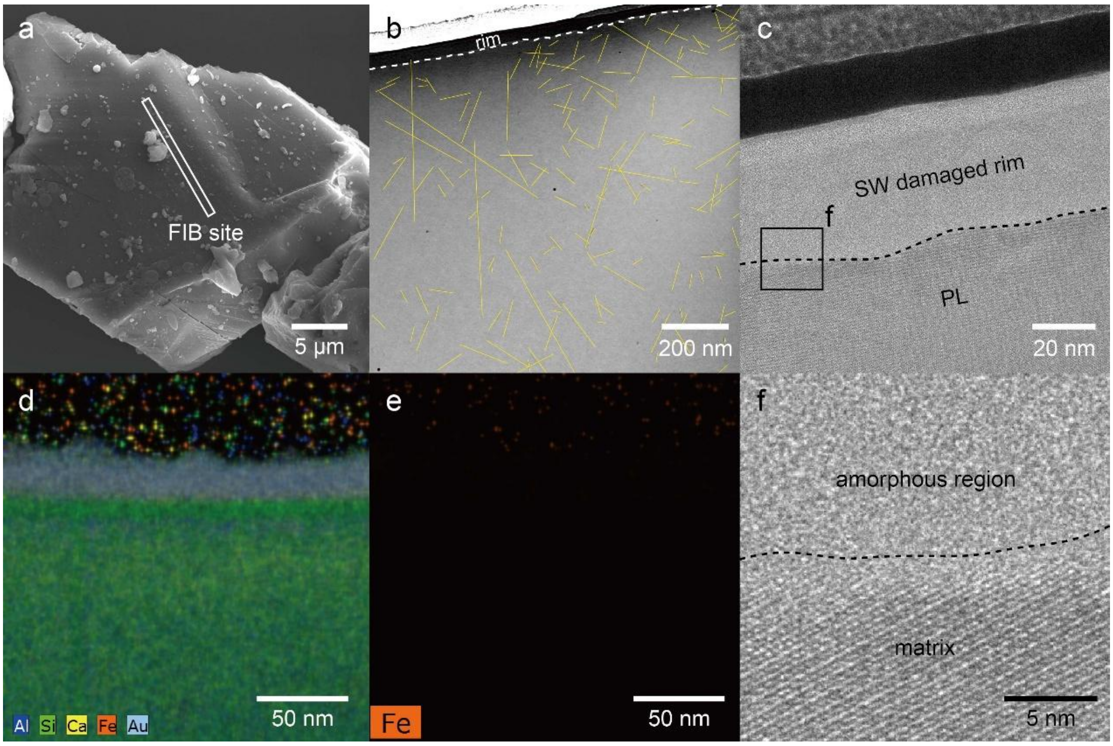

Fig. S2. (a) SE image of the studied CE5-PL2 grain. The white square indicates the FIB cross-section site. (b-c, f) STEM and HRTEM image of the FIB cross-section surface region. The dashed line represents the boundary between the solar wind damaged rim and the matrix. The solar energetic particle tracks within the matrix are marked by yellow solid lines. (d-e) Quantitative TEM-EDS composition maps (Au, Pt, Al, Si, Ca, Fe). The composition of the solar wind damaged rim is consistent with the matrix. SW, solar wind; PL, plagioclase.

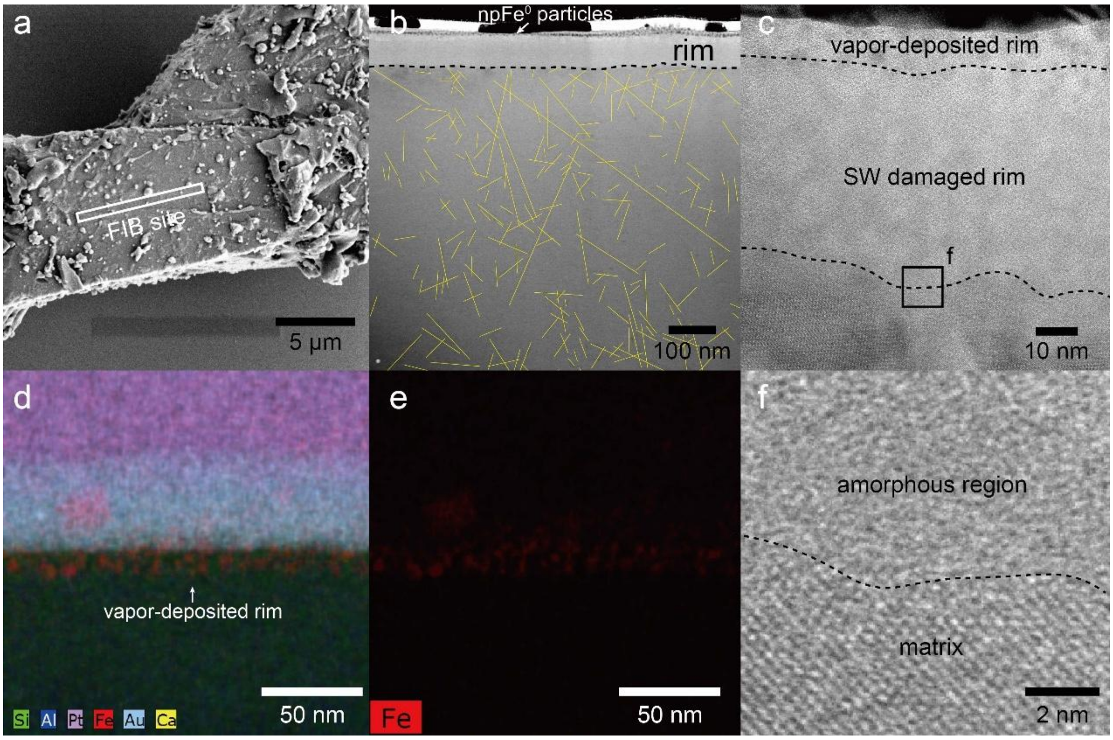

Fig. S3. (a) SE image of the studied CE5-PL3 grain. The white square indicates the FIB cross-section site. (b-c, f) STEM and HRTEM image of the FIB cross-section surface region. The black dashed line represents the boundary between the amorphous rim and the matrix. The rim includes both the vapor-deposited rim and the solar wind damaged rim. The solar energetic particle tracks within the matrix are marked by yellow solid lines. (d-e) Quantitative TEM-EDS composition maps (Au, Pt, Al, Si, Ca, Fe). The composition shows that the vapor-deposited rim is rich in iron, while the composition of the solar wind damaged rim is consistent with the matrix. SW, solar wind; PL, plagioclase.

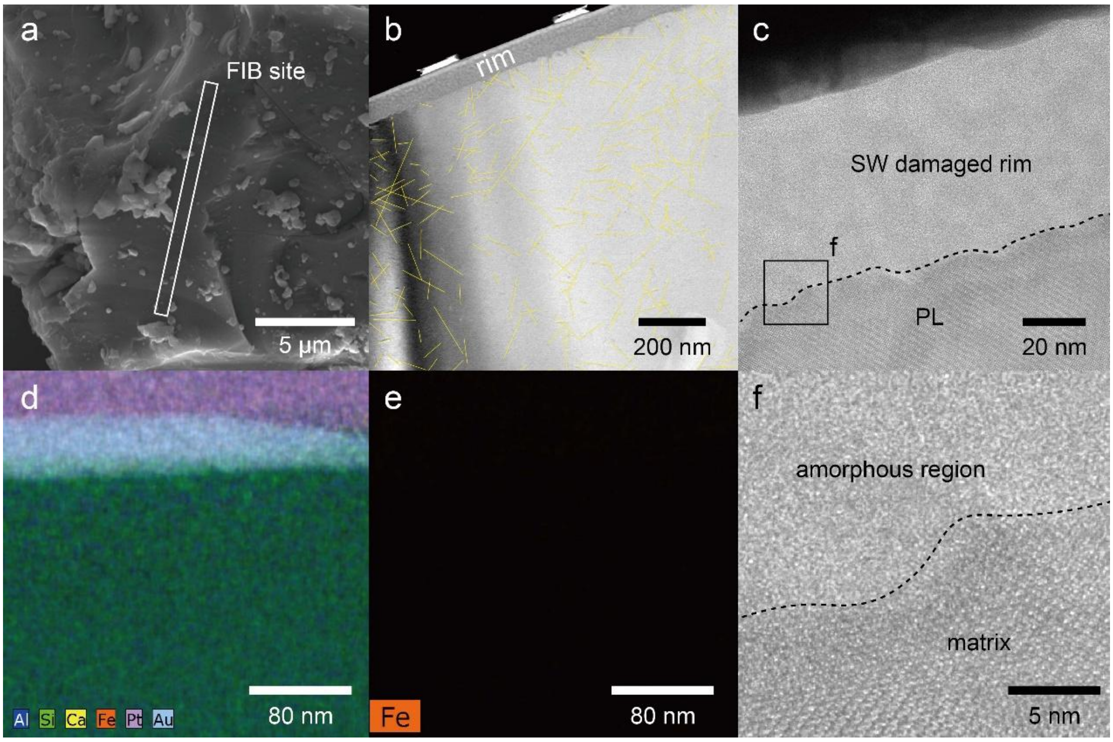

Fig. S4. (a) SE image of the studied CE5-PL4 grain. The white square indicates the FIB cross-section site. (b-c, f) STEM and HRTEM image of the FIB cross-section surface region. The black dashed line represents the boundary between the solar wind damaged rim and the matrix. The solar energetic particle tracks within the matrix are marked by yellow solid lines. (d-e) Quantitative TEM-EDS composition maps (Au, Pt, Al, Si, Ca, Fe). The composition of the solar wind damaged rim is consistent with the matrix. SW, solar wind; PL, plagioclase.

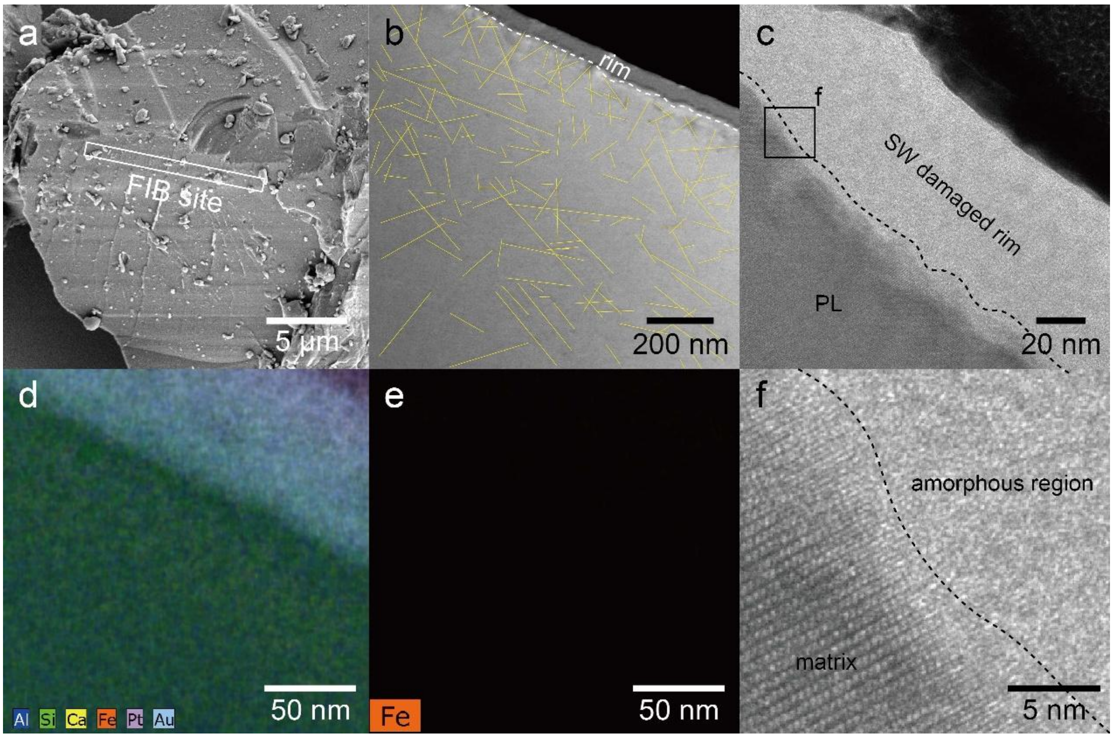

Fig. S5. (a) SE image of the studied CE5-PL5 grain. The white square indicates the FIB cross-section site. (b-c, f) STEM and HRTEM image of the FIB cross-section surface region. The dashed line represents the boundary between the solar wind damaged rim and the matrix. The solar energetic particle tracks within the matrix are marked by yellow solid lines. (d-e) Quantitative TEM-EDS composition maps (Au, Pt, Al, Si, Ca, Fe). The composition of the solar wind damaged rim is consistent with the matrix. SW, solar wind; PL, plagioclase.

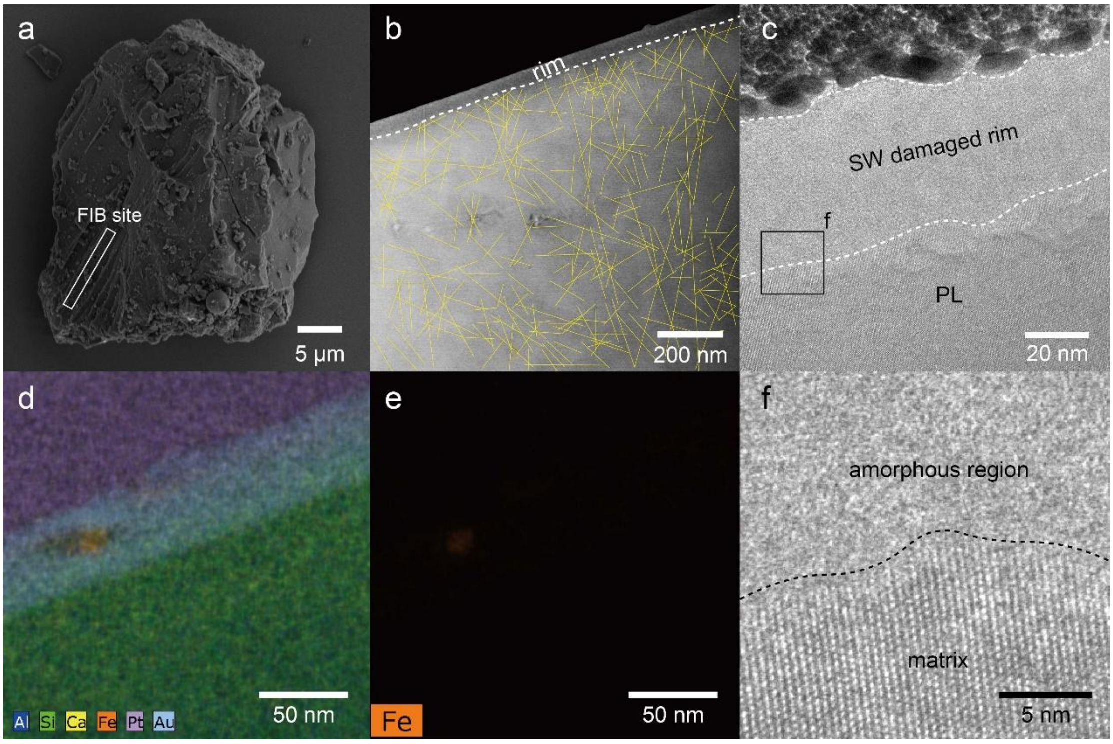

Fig. S6. (a) SE image of the studied CE5-PL6 grain. The white square indicates the FIB cross-section site. (b-c, f) STEM and HRTEM image of the FIB cross-section surface region. The dashed line represents the boundary between the solar wind damaged rim and the matrix. The solar energetic particle tracks within the matrix are marked by yellow solid lines. (d-e) Quantitative TEM-EDS composition maps (Au, Pt, Al, Si, Ca, Fe). The composition of the solar wind damaged rim is consistent with the matrix. SW, solar wind; PL, plagioclase.

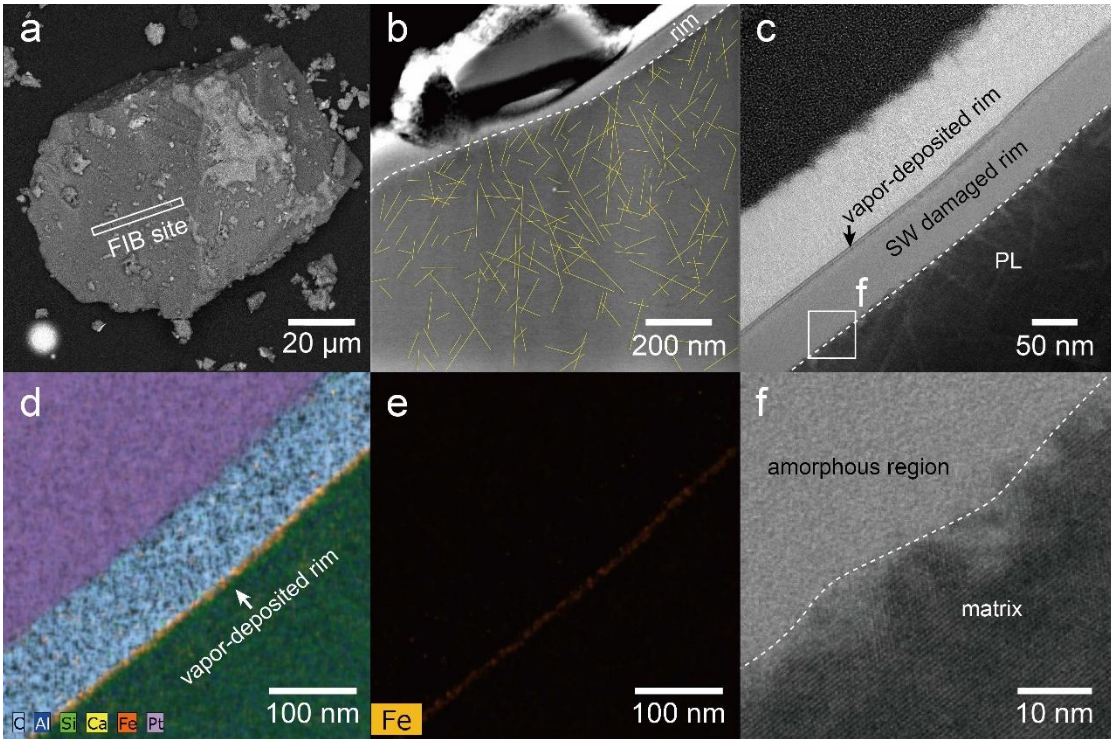

Fig. S7. (a) SE image of the studied CE5-PL7 grain. The white square indicates the FIB cross-section site. (b-c, f) STEM and HRTEM image of the FIB cross-section surface region. The white dashed line represents the boundary between the amorphous rim and the matrix. The rim includes both the vapor-deposited rim and the solar wind damaged rim. The solar energetic particle tracks within the matrix are marked by yellow solid lines. (d-e) Quantitative TEM-EDS composition maps (C, Pt, Al, Si, Ca, Fe). The composition shows that the vapor-deposited rim is rich in iron, while the composition of the solar wind damaged rim is consistent with the matrix. SW, solar wind; PL, plagioclase.

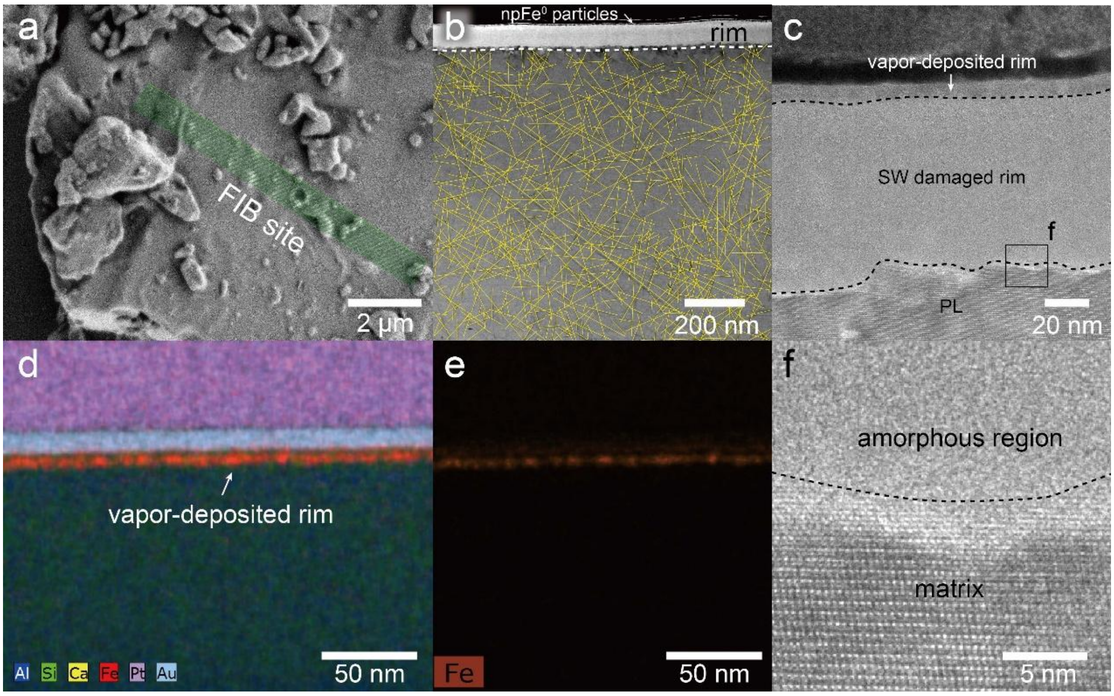

Fig. S8. (a) SE image of the studied CE6-PL1 grain. The green square indicates the FIB cross-section site. (b-c, f) STEM and HRTEM image of the FIB cross-section surface region. The dashed line represents the boundary between the amorphous rim and the matrix. The rim includes both the vapor-deposited rim and the solar wind damaged rim. The solar energetic particle tracks within the matrix are marked by yellow solid lines. (d-e) Quantitative TEM-EDS composition maps (Au, Pt, Al, Si, Ca, Fe). The composition shows that the vapor-deposited rim is rich in iron, while the composition of the solar wind damaged rim is consistent with the matrix. SW, solar wind; PL, plagioclase.

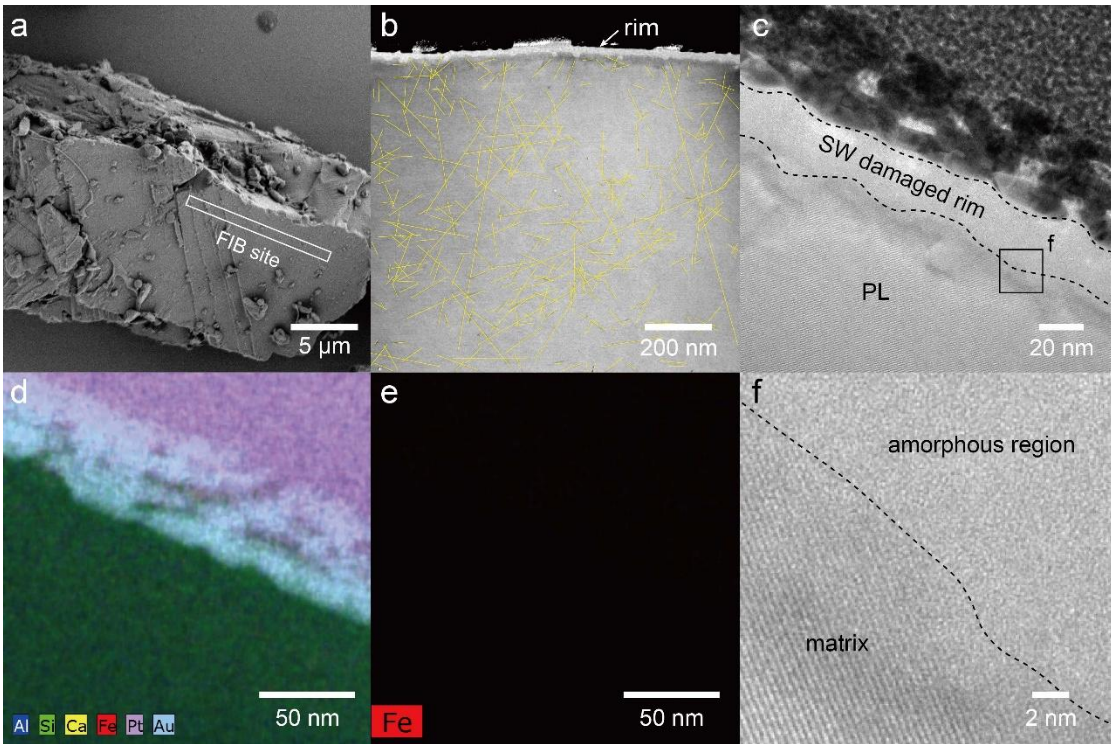

Fig. S9. (a) SE image of the studied CE6-PL2 grain. The white square indicates the FIB cross-section site. (b-c, f) STEM and HRTEM image of the FIB cross-section surface region. The black dashed line represents the boundary between the solar wind damaged rim and the matrix. The solar energetic particle tracks within the matrix are marked by yellow solid lines. (d-e) Quantitative TEM-EDS composition maps (Au, Pt, Al, Si, Ca, Fe). The composition of the solar wind damaged rim is consistent with the matrix. SW, solar wind; PL, plagioclase.

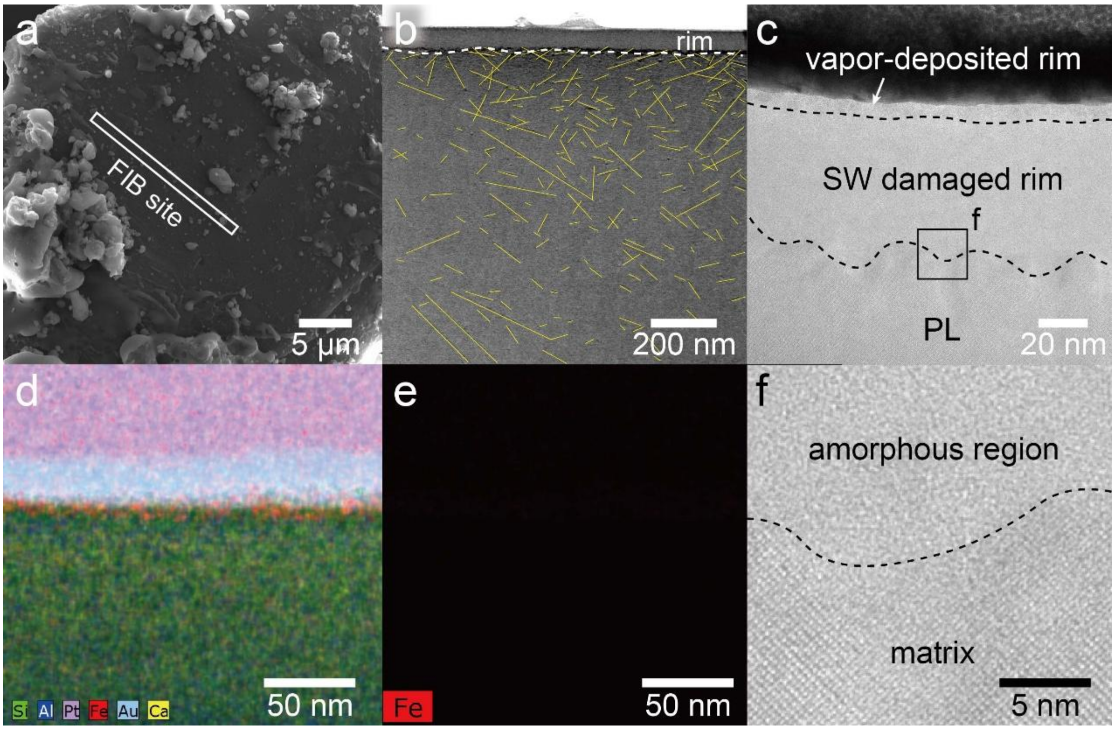

Fig. S10. (a) SE image of the studied CE6-PL3 grain. The white square indicates the FIB cross-section site. (b-c, f) STEM and HRTEM image of the FIB cross-section surface region. The dashed line represents the boundary between the amorphous rim and the matrix. The rim includes both the vapor-deposited rim and the solar wind damaged rim. The solar energetic particle tracks within the matrix are marked by yellow solid lines. (d-e) Quantitative TEM-EDS composition maps (Au, Pt, Al, Si, Ca, Fe). The composition shows that the vapor-deposited rim is extremely deficient in iron, while the composition of the solar wind damaged rim is consistent with the matrix. SW, solar wind; PL, plagioclase.

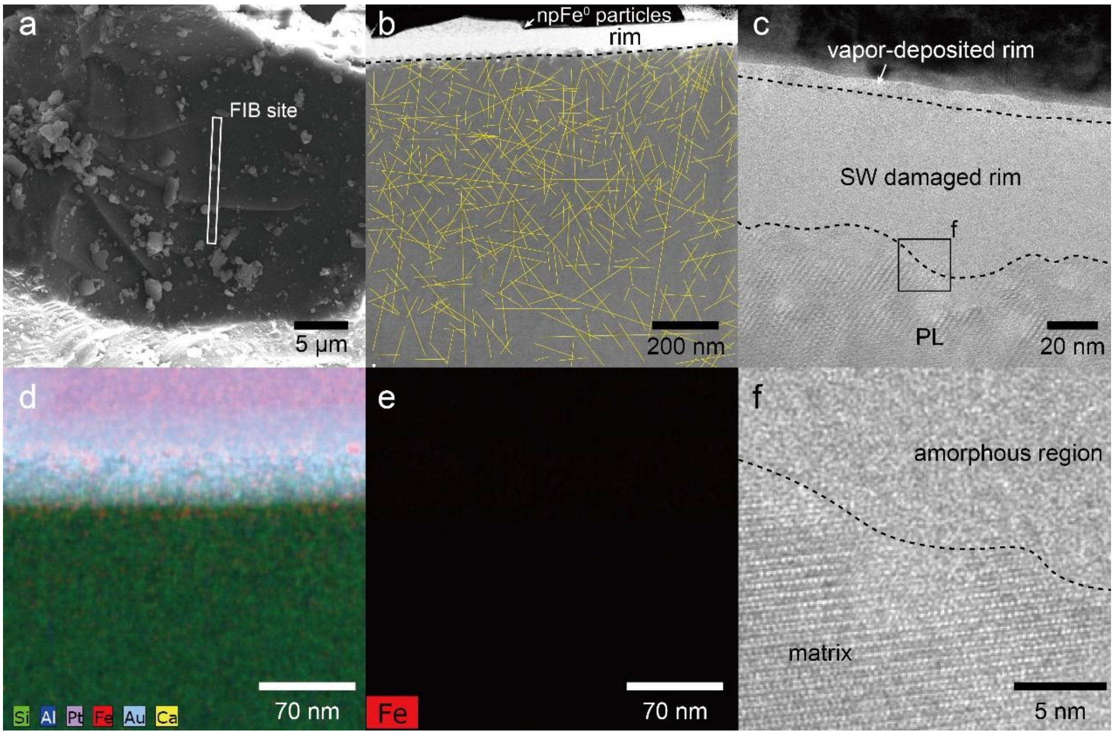

Fig. S11. (a) SE image of the studied CE6-PL4 grain. The green square indicates the FIB cross-section site. (b-c, f) STEM and HRTEM image of the FIB cross-section surface region. The black dashed line represents the boundary between the amorphous rim and the matrix. The rim includes both the vapor-deposited rim and the solar wind damaged rim. The solar energetic particle tracks within the matrix are marked by yellow solid lines. (d-e) Quantitative TEM-EDS composition maps (Au, Pt, Al, Si, Ca, Fe). The composition shows that the vapor-deposited rim is extremely deficient in iron, while the composition of the solar wind damaged rim is consistent with the matrix. SW, solar wind; PL, plagioclase.

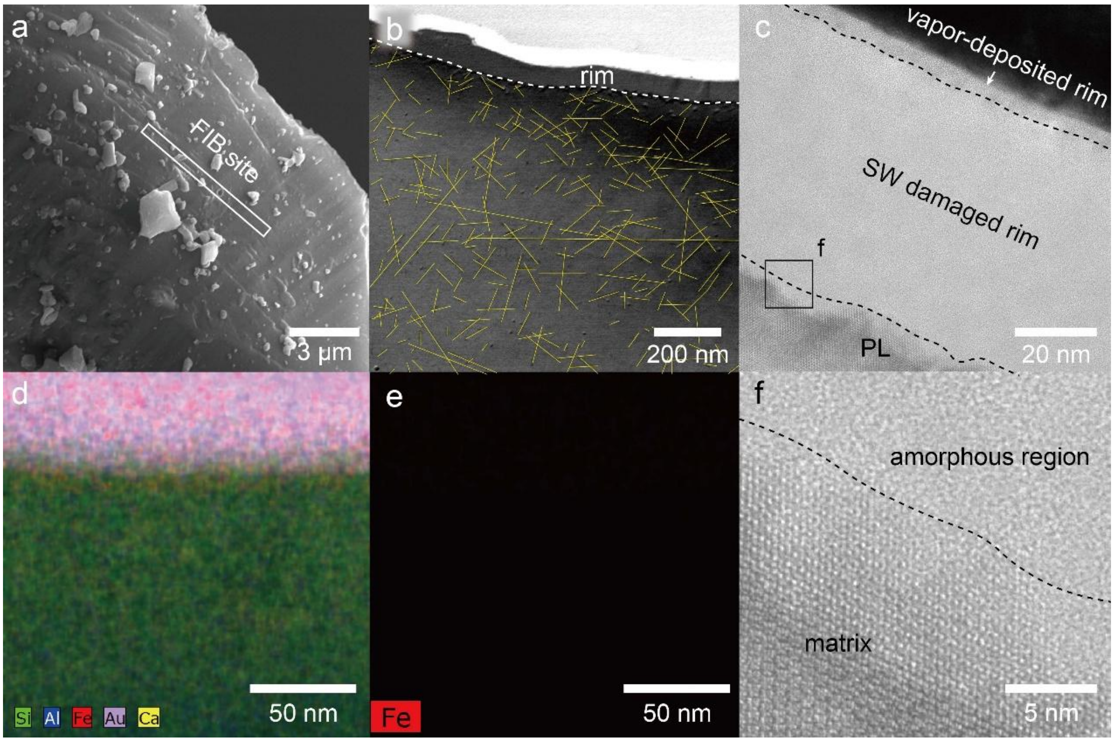

Fig. S12. (a) SE image of the studied CE6-PL5 grain. The green square indicates the FIB cross-section site. (b-c, f) STEM and HRTEM image of the FIB cross-section surface region. The dashed line represents the boundary between the amorphous rim and the matrix. The rim includes both the vapor-deposited rim and the solar wind damaged rim. The solar energetic particle tracks within the matrix are marked by yellow solid lines. (d-e) Quantitative TEM-EDS composition maps (Au, Pt, Al, Si, Ca, Fe). The composition shows that the vapor-deposited rim is extremely deficient in iron, while the composition of the solar wind damaged rim is consistent with the matrix. SW, solar wind; PL, plagioclase.

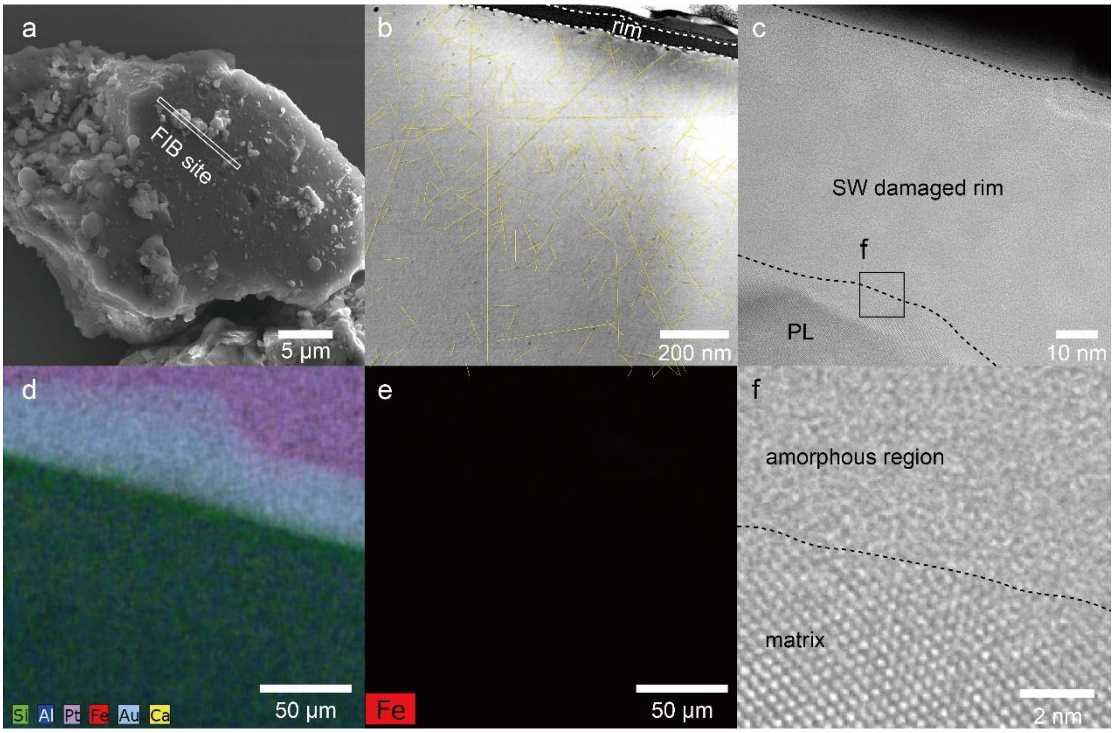

Fig. S13. (a) SE image of the studied CE6-PL6 grain. The white square indicates the FIB cross-section site. (b-c, f) STEM and HRTEM image of the FIB cross-section surface region. The dashed line represents the boundary between the solar wind damaged rim and the matrix. The solar energetic particle tracks within the matrix are marked by yellow solid lines. (d-e) Quantitative TEM-EDS composition maps (Au, Pt, Al, Si, Ca, Fe). The composition of the solar wind damaged rim is consistent with the matrix. SW, solar wind; PL, plagioclase.

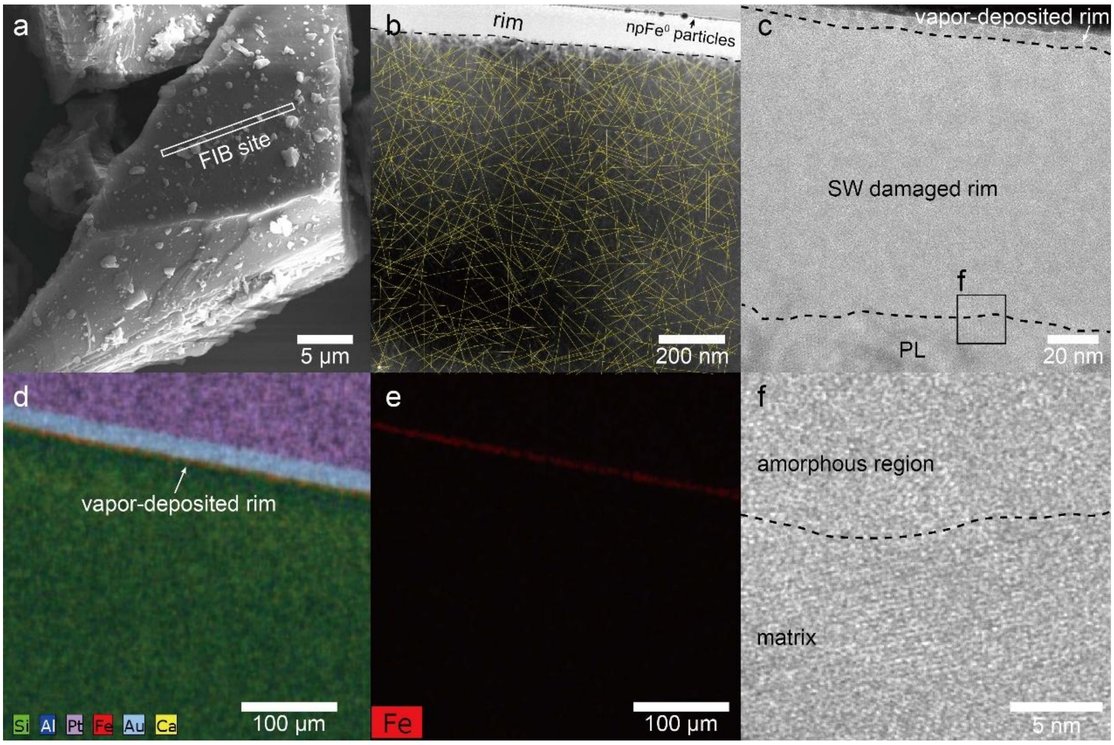

Fig. S14. (a) SE image of the studied CE6-PL7 grain. The green square indicates the FIB cross-section site. (b-c, f) STEM and HRTEM image of the FIB cross-section surface region. The black dashed line represents the boundary between the amorphous rim and the matrix. The rim includes both the vapor-deposited rim and the solar wind damaged rim. The solar energetic particle tracks within the matrix are marked by yellow solid lines. (d-e) Quantitative TEM-EDS composition maps (Au, Pt, Al, Si, Ca, Fe). The composition shows that the vapor-deposited rim is rich in iron, while the composition of the solar wind damaged rim is consistent with the matrix. SW, solar wind; PL, plagioclase.

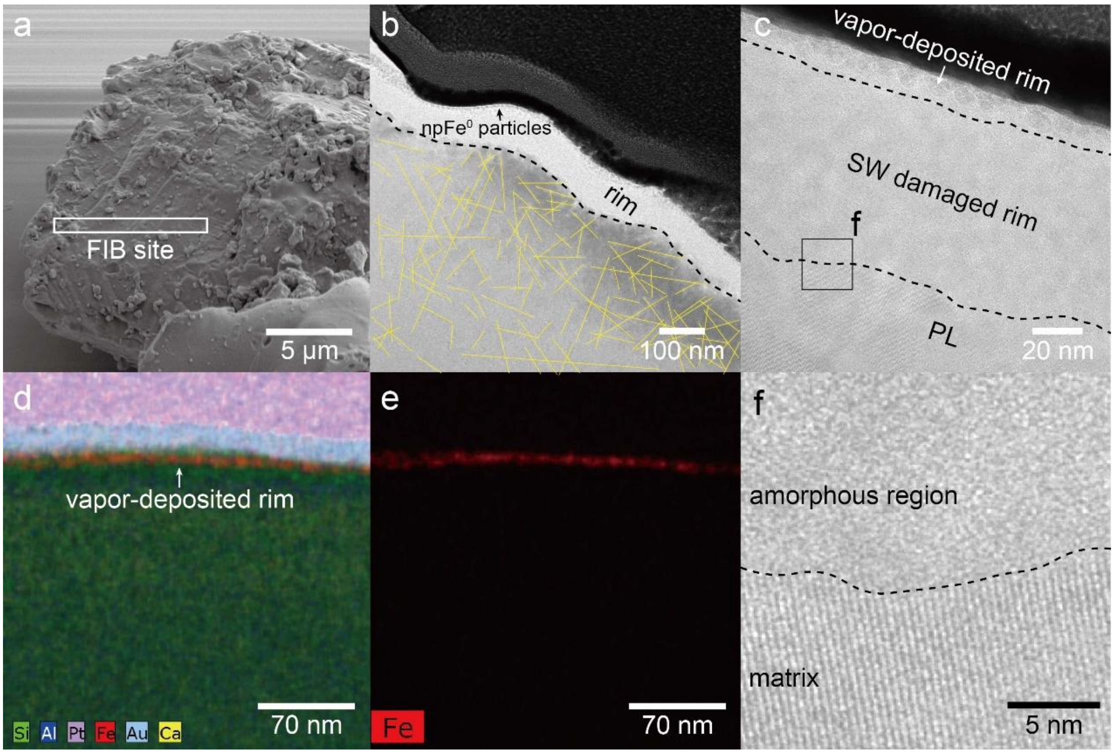

Fig. S15. (a) SE image of the studied CE6-PL8 grain. The green square indicates the FIB cross-section site. (b-c, f) STEM and HRTEM image of the FIB cross-section surface region. The black dashed line represents the boundary between the amorphous rim and the matrix. The rim includes both the vapor-deposited rim and the solar wind damaged rim. The solar energetic particle tracks within the matrix are marked by yellow solid lines. (d-e) Quantitative TEM-EDS composition maps (Au, Pt, Al, Si, Ca, Fe). The composition shows that the vapor-deposited rim is rich in iron, while the composition of the solar wind damaged rim is consistent with the matrix. SW, solar wind; PL, plagioclase.

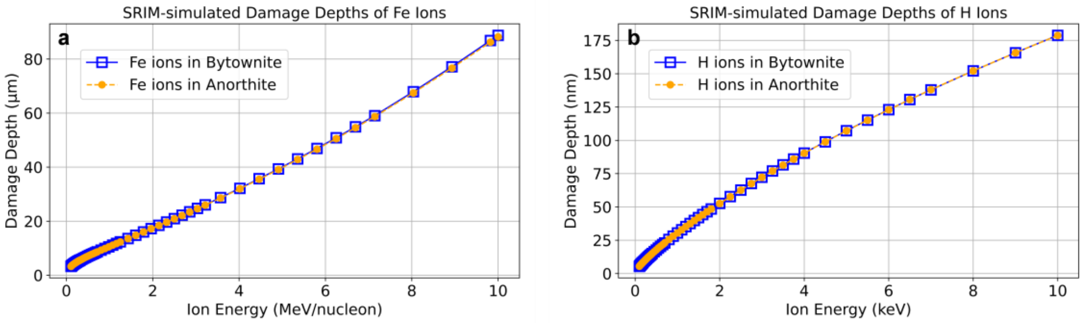

Fig. S16. SRIM-simulated damage depths for (a) Fe and (b) H ions implanted into bytownite ($Na_{0.3}Ca_{0.7}Al_{1.3}Si_{2.7}O_8$, density = 2.72 g/cm³) and anorthite ($CaAl_2Si_2O_8$, density = 2.75 g/cm³). For each ion species and implantation energy, the resulting damage depths are nearly identical between the two minerals, indicating negligible dependence on target composition.
